# Patient safety: adverse effects of Clozapine and their management at the Psychiatric Hospital Ghrasia in Yogyakarta, Indonesia

**DOI:** 10.25122/jml-2023-0145

**Published:** 2023-11

**Authors:** Bangunawati Rahajeng, Betha Candra Sari, Nurul Maziyyah

**Affiliations:** 1 Department of Pharmacology & Clinical Pharmacy, School of Pharmacy, Faculty of Medicine and Health Sciences, Universitas Muhammadiyah Yogyakarta, Indonesia; 2 School of Pharmacy, Faculty of Medicine and Health Sciences, Universitas Muhammadiyah Yogyakarta, Indonesia

**Keywords:** clozapine, psychiatric hospital, side-effect, schizophrenia

## Abstract

Clozapine is an atypical antipsychotic indicated for treating patients resistant to antipsychotic therapy when other medications in this class have no therapeutic effect. Clozapine has greater efficacy but more side effects than other atypical antipsychotics (e.g., agranulocytosis, seizures, sedation, and weight gain). Side effects can cause discomfort to patients and can affect patient compliance, interfering with therapeutical outcomes. This study aims to identify the side effects of clozapine use in patients at the Psychiatric Hospital Grhasia, in Yogyakarta, Indonesia. Using a cross-sectional design, this research provides a retrospective descriptive analysis using medical records of patients at the Psychiatric Hospital Ghrasia Yogyakarta. Inpatient and outpatient medical records of patients who had received a clozapine prescription from January to December 2019 were analyzed. In the 336 patients that met the inclusion criteria, the incidence of side effects from clozapine use was 16.07% (95%, CI:2.2-4.1), with the most frequent being dizziness, vomiting, diarrhea, and hypersalivation. There was no effect of age, gender, profession, or patient's disease on their incidence. The management of side effects is classified into three aspects: the drug is stopped, the patient is given additional therapy, and the patient is not given therapy. The results showed that the use of clozapine at the Psychiatric Hospital Ghrasia Yogyakarta was relatively safe. Identification of side effects is necessary to determine the follow-up treatment.

## INTRODUCTION

Clozapine is the first atypical antipsychotic agent with high effectiveness and few extrapyramidal side effects. Giving clozapine, in general, can improve attention and verbal ability. Atypical antipsychotics, such as clozapine, have a higher anti-inflammatory and cytokine-boosting effect, including interleukin-1 alpha (IL-1α), IL-4, IL-6, IL-10, tumor necrosis factor alpha (TNF-α), interferon gamma (IFN-γ), and tumor growth factor beta 1 (TGF-β1), compared to first-generation (typical) antipsychotics. Clozapine is indicated for patients resistant to antipsychotic therapy (when other antipsychotics have failed) [1, 2]. It presents the highest incidence of weight gain and has a strong sedative side effect but a very low incidence of extrapyramidal side effects [3].

Clozapine has great efficacy but has more side effects than other atypical antipsychotics (e.g., agranulocytosis, seizures, sedation, and weight gain). It is associated with a high risk of low white blood cell levels, resulting in death; to reduce this risk, it is necessary to have regular blood tests [4, 5].

The aim of this study was to identify the side effects of clozapine in patients at Gharsia Psychiatric Hospital and methods to address them.

## MATERIAL AND METHODS

This cross-sectional research uses descriptive analysis to offer insight into retrospectively collected medical record data of patients who received a clozapine prescription at the Grhasia in 2019. All patients (inpatient and outpatient) who received clozapine therapy from January to December 2019 were eligible for inclusion. Data included patient characteristics, diagnosis, treatment history, complaints, and condition at discharge. The researchers excluded incomplete and illegible medical records, such as patients who did not finish therapy due to a discharge against medical advice or death. The data of 336 patients met the inclusion criteria.

The incidence rate of side effects experienced by patients is presented as a percentage. We use the Naranjo algorithm (Table 1) to analyze the causality of the side effect to make sure that the side effect is due to clozapine. The Naranjo algorithm contains ten questions to identify the causal relationship between drugs suspected of causing adverse effects.

**Table 1:** Adverse Drug Reaction Probability Score

No	Questionnaire	Yes	No	Don’t know	Score
1.	Are there previous conclusive reports on this reaction?	+1	0	0	
2.	Did the adverse event appear after the suspected drug was administered?	+2	-1	0	
3.	Did the adverse reaction improve when the drug was discontinued or a specific antagonist was administered?	+1	-1	0	
4.	Did the adverse reaction reappear when the drug was readministered?	+2	-1	0	
5.	Are there alternative causes (other than the drug) that could on their own have caused the reaction?	-1	+2	0	
6.	Did the reaction reappear when the placebo was given?	-1	0	0	
7.	Was the drug detected in the blood (or other fluids) in the concentrations known to be toxic?	+1	0	0	
8.	Was the reaction more severe when the dose was increased, or less severe when the dose was decreased?	+1	0	0	
9.	Did the patient have a similar reaction to the same or similar drugs in any previous exposure?	+1	0	0	
10.	Was the adverse event confirmed by any objective evidence?	+1	0	0	

The first question is answered by searching the literature/journals. Since this study was retrospective, the second, third, fourth, fifth, ninth, and tenth questions were answered using medical record data. The sixth, seventh, and eighth questions were scored 0 (don't know).

## RESULTS

In this study, the recorded data of 336 patients who received a prescription for clozapine between January and December 2019 underwent multivariate analysis to identify the variables associated with an increased risk of side effects under treatment with Clozapine. The patient characteristics are presented in Table 2.

**Table 2:** Patient characteristics

Characteristics	Total (n)	Percentage (%)	p-value	95% CI	
				Lower	Upper
**Gender**					
Female	191	56.85	0.115	0.890	2.921
Male	145	43.15			
**Age (years old)**					
≤55	293	87.20	0.484	0.608	2.856
>55	43	12.78			
**Occupation**					
Unemployed	207	61.60	0.168	0.838	2.764
Employed	129	38.40			
**Diagnosis**					
Schizophrenia	239	71.13	0.838	0.575	1.980
Other diagnoses	97	28.87			
**Total**	**336**	**100**			

At Ghrasia, clozapine is prescribed for several mental disorders such as schizophrenia, mood disorders, intellectual disability, etc. Of the 336 patients meeting the inclusion criteria, 54 experienced side effects, such as headaches, vomiting, diarrhea, hypersalivation, nausea, and cramps. Table 3 displays the incidence rate of side effects.

**Table 3:** Incidence of side effects in patients who received Clozapine prescription

Side-Effects	Number (n)	%
Dizziness	6	1.79
Vomiting	6	1.79
Diarrhea	6	1.79
Hypersalivation	6	1.79
Nausea	5	1.49
Seizure	4	1.19
Fever	4	1.19
Rigidity	3	0.89
Weight gain	3	0.89
Tachycardia	2	0.6
Constipation	2	0.6
Tremor	2	0.6
Hypotension	1	0.3
Sedation (drowsiness)	1	0.3
Dyspepsia	1	0.3
Urine Retention	1	0.3
Leukocytosis	1	0.3
**Total**	**54**	**16.07**

The incidence of side effects was determined based on the Naranjo algorithm, which is a questionnaire designed to determine whether an adverse effect is caused by the suspected drug or by other factors [6]. The results of the analysis are presented in Table 4.

**Table 4:** The results of the Naranjo score on Clozapine

Side-Effects	Algoritm Naranjo Score	Explanation
Dizziness	3	Possible
	4	Possible
	6	Probable
	3	Possible
	5	Probable
	4	Possible
Tachycardia	3	Possible
	4	Possible
Constipation	4	Possible
	3	Possible
Nausea	4	Possible
	5	Probable
	3	Possible
	6	Probable
	4	Possible
Vomiting	4	Possible
	5	Probable
	4	Possible
	3	Possible
	5	Probable
	6	Probable
Sedation/ Drowsiness	6	Probable
Weight gain	5	Probable
	6	Probable
	5	Probable
Hypotension	4	Possible

Patients experiencing side effects received treatment from the hospital; the management of clozapine side effects can be seen in Table 5.

**Table 5:** Management of Clozapine side effects in patients at Ghrasia Hospital Yogyakarta

Side-effect management	Number	%
Added therapy	39	72.2
No therapy	13	24.1
Drugs stopped	2	3.7
**Total**	**54**	**100**

## DISCUSSION

Patients receiving clozapine prescriptions were predominantly male. According to Novitayani, the appearance of symptoms often occurs at the age of 15-25 years in the male gender (53.12%), with symptoms only appearing in three female patients (9.37%) in the same age group. Women (average age 30 years) tend to develop schizophrenia at an older age than men (mean age 25 years) [7].

Socially, men have a higher life pressure than women, and this can trigger stress. Women also have more estrogen in their bodies, which affects dopamine activity in the nucleus accumbens by inhibiting its release. The increase in the number of dopamine receptors in the caudate nucleus, accumbens, and putamen is one of the etiologies of schizophrenia. This hormone's neuroprotective effect will indirectly impact the onset and course of schizophrenia in women [8, 9].

Schizophrenia can arise from a young age, making it difficult for patients to adapt to their environment. This, in turn, affects their ability to maintain a job, leading to increased stress and feelings of helplessness. This condition is associated with high levels of stress hormones (catecholamines). Meanwhile, working people have a sense of optimism about the future and greater enthusiasm for life [10].

Most of the patients admitted at Grhasia in January-December 2019 were diagnosed with schizophrenia, including a total of 239 patients of the 336 patients in this study (71%). This is in agreement with the study previously conducted by Novitayani, which stated that the number of schizophrenia cases in Aceh Province was higher than other mental disorders [7]. Schizophrenia is a common mental disorder characterized by disturbances and peculiarities of thought, perception, emotion, movement, and behavior [7, 11]. Schizophrenia does not occur by itself; many factors play a role in its incidence, including genetic, biological, biochemical, psychosocial, and socioeconomic factors, as well as stress and drug abuse [12].

The multivariate test conducted on patients’ sociodemographics found that age, gender, occupation, and diagnosis did not affect the incidence of side effects. Several findings differ from the review by Zhang *et al*., stating that women are more at risk for side effects. While some authors found that age is also a risk factor for side effects, especially at <50, some find no relationship between age and the incidence of side effects [13]. Research conducted by Iqbal *et al*. stated that specific characteristics influenced the occurrence of specific side effects from clozapine. For example, gender influences backache, constipation, diarrhea, fatigue, feeling sick, hyperprolactinemia, and stomach pain, while age affects the occurrence of agitation, fatigue, general sickness, sedation, shaking, tachycardia, and weight gain [14]. Patel *et al*. conducted a study at the psychiatric department of an Indian hospital showcasing that schizophrenia co-occurs with many side effects. However, Patel *et al*. did not analyze the association between diagnosis and adverse events. In contrast, the review by Zhou *et al*. found comorbid factors and central nervous system agents as risk factors for side effects [15, 16]. Kitagawa *et al*. stated that the duration of clozapine administration, dose, and plasma concentration did not affect the incidence of clozapine side effects. In that study, plasma concentrations of clozapine were higher in the group that experienced side effects, although this was not statistically significant [17]. However, in our study, we did not obtain data on clozapine plasma concentrations because, in Indonesia, blood levels of the drug are rarely monitored except under particular conditions.

Not all patients experience drug side effects. If side effects occur, they occur differently for each patient. Several factors, such as individual conditions, comorbidities, and combinations of antipsychotic therapy influence side effects. The side effects of second-generation antipsychotics are related to the interaction of these drugs with multiple receptors, like muscarinic, histaminic, and alpha-adrenergic receptors. Extrapyramidal side effects include tremors, hypersalivation, stiffness, and restlessness which are mainly caused as a result of a combination of typical antipsychotic drugs with severe symptoms. However, extrapyramidal effects may occur with the use of atypical antipsychotics with milder symptoms, as this medication generally has a high affinity to block dopamine receptors. Atypical antipsychotics can also cause extrapyramidal affections, due to blocking dopamine in the nigrostriatal area [18, 19].

Research by Yulianty *et al*. indicated that antipsychotic combination therapy was the most widely used therapy for schizophrenic patients (90.6%), with the most widely used drug being haloperidol-clozapine (26.06%). The side effects that occurred in 59 patients were extrapyramidal syndrome (98.3%); orthostatic hypotension (86.4%); anticholinergic effects (76.3%); sedation (44.1%); nausea/vomiting (27.1%); diarrhea (27.1%); insomnia (16.9%); no appetite (10.2%); itchy redness (6.8%); anorexia (5.1%); frequent urination (5.1%); decreased consciousness (1.7%); shortness of breath and cough (1.7%); a decrease in hemoglobin value (1.7%); an increase in Aspartate aminotransferase (1.7%); an increase in Alanine aminotransferase (1.7%); and nasal mucus (1.7%) [20].

In this study, the side effects of clozapine were determined based on the Naranjo algorithm. Several factors that influence their incidence include individual differences in tolerating the side effects of each drug. The more combinations of drugs used, the greater the risk of side effects will be. The most widely used drugs in combination are clozapine, risperidone, and trihexyphenidyl (THP). Clozapine works in the same way as risperidone. It reduces positive symptoms and stabilizes effective symptoms by blocking serotonin 2A receptors, causing an increase in dopamine release in brain regions, and has the lowest risk of extrapyramidal side effects. First and second-generation antipsychotics can cause side effects in the form of sedation, autonomic disturbances, extrapyramidal disorders, and disturbances in the metabolic system [19, 21].

The Naranjo assessment was carried out on clozapine and other drugs with the same incidence of side effects as risperidone to exclude the risk of side effects due to other medications. The results showed 15 patients with a score of 1-4 (possible) and 11 patients with a score of 5-8 (probable), indicating that it was most likely that 11 people experienced clozapine side effects. A score of 3-4 indicated that the patient's complaint was likely to be a side effect event, with the Naranjo algorithm score on clozapine showing higher results than other drug scores. Thus, patients who had a Naranjo algorithm score of 3-4 were included when calculating the incidence of side effects. Similarly, a Naranjo algorithm score of 5-6 showed that the patient's possible complaint was a side effect of the suspected drug, and the use of clozapine showed a higher score than other drug scores.

In a systematic review conducted by Lobos *et al*. the side effects of clozapine were neutropenia, hypersalivation, sedation, seizures, and weight gain. These side effects outweigh the similar side effects of risperidone, olanzapine, and quetiapine. A more recent review by Wagner *et al*. mentioned the incidence of agranulocytosis, constipation, and heart affections, such as arrhythmias and cardiomyopathy, with clozapine use [1, 22].

The management of side effects is classified in three ways: the drug is stopped, the patient is given additional therapy, or the patient is not given therapy. Out of the total incidence of side effects, clozapine therapy was discontinued in two patients (3.7%), while 39 patients were given additional therapy (72.2%), and 13 patients were not given therapy (24.1%).

Treatment using clozapine was stopped in two patients, who experienced hypersalivation and limb weakness. One patient experienced sedation or drowsiness and received treatment to decrease the dose of clozapine. Some patients receive additional therapy according to the side effects or complaints experienced. For example, the side effects of dizziness or headache were experienced by four patients, who were given additional therapy of paracetamol 500 mg if necessary. Paracetamol was also given to three patients who presented fever. Nausea was treated by additional therapy of domperidone three times a day, and antacids if necessary. Vomiting was experienced by some patients, who were given therapy in the form of domperidone three times a day if necessary, or diphenhydramine injection, which was adjusted to the patient's condition. In patients who experienced seizures, additional therapy was given as a combination of phenobarbital, phenytoin, and caffeine. Diarrhea was experienced by several patients who received additional therapy in the form of oral rehydration solution and attalpugite. Cotrimoxazole was given to one patient to treat an infection.

Drug side effects need to be addressed as they are likely to impact therapy failure, increase severe disease, and decrease medication adherence. However, in this study, some patients who experienced side effects were not given any therapy. The most common side effect that was not given any additional therapy was weight gain, as this does not cause dangerous conditions for the patient. Wagner *et al*. mentioned in their review article that something can be done to overcome side effects. Metformin can be given to patients with weight gain, as it has been shown to be superior to placebo in weight loss. Aripiprazole is effective in terms of short-term weight loss and reduction of lipid levels with little effect. Topiramate can be used to treat weight loss. Furthermore, there are no specific guidelines for constipation and heart problems. [22].

A total of 232 patients (69.05%) were discharged with a better condition status and were asked to have regular medical control. Meanwhile, 103 patients (30.65%) were discharged with improved condition status, and one patient’s condition (0.30%) worsened.

The treatment phases in schizophrenia consists of an acute phase, a stabilization phase, and a maintenance phase. Specifically, it can be examined by identifying a clear psychotic picture (delusions, hallucinations, thinking disorders, etc.). The stabilization and maintenance phase is carried out to improve the recovery process and ensure that symptom control continues [23]. Therefore, at the Psychiatric Hospital Gherasia in Yogyakarta, most patients were discharged with control conditions, because therapy was needed during the maintenance and stabilization phase. The research was limited by incomplete data, lacking variables such as length of therapy, and comorbid factors, which might be risk factors for side effects. However, this research has been anticipated by only using complete medical record data.

Based on the results, many aspects should be considered with regard to this medication, including more intense monitoring of side effects, which requires interprofessional cooperation, especially between doctors, pharmacists, and nurses. Some side effects that have not previously been addressed also require further attention. Moreover, as this study was carried out retrospectively, the is that it did not identify side effects that required laboratory tests, such as agranulocytosis, complete blood count, or electrocardiography.

## CONCLUSION

The use of clozapine at the Psychiatric Hospital Ghrasia Yogyakarta was relatively safe. The incidence of side effects from clozapine use was 16.07% (95% CI:2.2-4.1), out of 336 patients. The most encountered manifestations were dizziness, vomiting, diarrhea, and hypersalivation. Identification of side effects is necessary to achieve optimal therapy. Pharmacists can play their role by counseling on the side effects that may occur with the use of clozapine. Moreover, education should include guidance on managing situations where side effects occur.

## Special thanks

The authors would like to thank the institute for research, publication, and community services Universitas Muhammadiyah Yogyakarta for funding this research, and Psychiatric Hospital Ghrasia Yogyakarta for permitting this research to be conducted.
